# Precis of *Evidential Pluralism in the Social Sciences*

**DOI:** 10.1007/s44204-025-00301-y

**Published:** 2025-07-10

**Authors:** Yafeng Shan, Jon Williamson

**Affiliations:** 1 Center for Philosophy of Science and Division of Humanities, The Hong Kong University of Science and Technology, Hong Kong, Hong Kong; 2 Department of Philosophy, University of Manchester, Manchester, UK

**Keywords:** Evidential pluralism, Causality, Causation, Social sciences, Methodology, Causal enquiry

## Abstract

Evidential Pluralism is an emerging philosophical theory of how to establish and evaluate causal claims. Shan & Williamson (2023) apply Evidential Pluralism across the social sciences. This article provides a concise overview of the book.

Evidential Pluralism is an epistemological account of causal enquiry, consisting of two theses: object pluralism and study pluralism.

- Object pluralism: In order to establish the claim that A causes B, one normally needs to establish an appropriate correlation between A and B and an appropriate mechanism complex linking A to B.
- Study pluralism: When assessing a causal claim, one ought to consider association studies and mechanistic studies, where available.

It should be emphasised that both theses are normative, in the sense that they provide an account of what scientists ought to do in order to establish a causal claim, rather than an account of what scientists actually do when they try to establish a causal claim. The central argument of Shan and Williamson (2023) is that Evidential Pluralism can be fruitfully applied to the social sciences.

The book begins with an introduction to Evidential Pluralism. In Chapter 1, we develop Evidential Pluralism as a general theory of causal enquiry. We elaborate the two main theses: object pluralism and study pluralism. We explain the relevant terminology in order to avoid potential misconceptions. We also provide motivation for the theory, explaining why Evidential Pluralism is plausible. We sketch its previous successful application to the biomedical sciences and clarify the ways in which Evidential Pluralism can be used. While Evidential Pluralism is an epistemological theory concerned with how to establish and assess causal claims, we outline one particular philosophical theory of the nature of causality that coheres well with Evidential Pluralism, namely, epistemic causality.

Although Evidential Pluralism arose out of a thesis of Russo and Williamson that was put forward in 2007, the interplay between correlation and mechanisms attracted some interest in the nineteenth and twentieth centuries. In Chapter 2, we explore some views that might be thought of as historical precursors of Evidential Pluralism, including the approaches of the French physiologist Claude Bernard, the English biologist W.F.R. Weldon and the American sociologist John Goldthorpe. We also examine some differences between Evidential Pluralism and analytic sociology, and some differences between Evidential Pluralism and Roy Bhaskar’s critical realism.

Evidential Pluralism was previously applied to produce an extension of evidence-based medicine (EBM) that systematically scrutinises mechanistic studies alongside the association studies that are the focus of orthodox EBM. The resulting approach is called EBM + . In Chapter 3, we argue for an analogous move in the area of evidence-based policy. We show that Evidential Pluralism leads to a new account of evidence-based policy (EBP), which we call EBP + . EBP + provides the capability to assess mechanistic studies alongside the association studies that are the focus of present-day evidence-based policy assessment. This approach can lead to better-informed judgements of the effectiveness of social interventions, and thus to better social policy. We compare this new account to related approaches, such as realist evaluation.

In Chapter 4, we argue that Evidential Pluralism provides new philosophical foundations for mixed methods research in the social sciences. Mixed methods research has become increasingly popular in the social sciences. Meanwhile, there has been an ongoing debate about the philosophical foundations of mixed methods research. We sketch the main approaches to the foundations of mixed methods research and note certain limitations. We then argue that the account of causal enquiry at the heart of Evidential Pluralism requires a thorough consideration of both quantitative and qualitative methods and so provides strong motivation for mixed methods research in causal enquiry. Evidential Pluralism can also provide practical guidance on how to integrate quantitative and qualitative studies.

Despite its merits, the application of Evidential Pluralism to the social sciences has been contested. In Chapter 5, we defend Evidential Pluralism by responding to four potential objections to Evidential Pluralism. The first two objections concern the necessity and sufficiency of evidence of correlation and of mechanisms in establishing and assessing causal claims: an objection that establishing correlation and mechanism is not sufficient to establish causation, and an objection that establishing both correlation and mechanism is not necessary for establishing causation. Next, we address an objection that appeals to causal pluralism—the view that there are multiple concepts of cause in use in the social sciences. Finally, we respond to concerns about how to define ‘mechanism’ in the social sciences.

In the remaining chapters of the book, we show how Evidential Pluralism can be fruitfully applied to a variety of social sciences. In Chapter 6, we argue that applying Evidential Pluralism to sociology brings several advantages. Firstly, we show that Evidential Pluralism can shed light on the use of evidence in causal enquiry in sociology by means of two examples: one involving the connection between socioeconomic status and health, and one concerning the link between family background and educational attainment. We then show that Evidential Pluralism motivates two important approaches to the methodology of causal enquiry in sociology, namely that of Goldthorpe (2001) and that of Morgan and Winship (2015). We conclude that Evidential Pluralism provides a unified approach to causal enquiry in sociology.

The question whether Evidential Pluralism can be fruitfully applied to economics has already attracted some attention. Some are optimistic, while others are sceptical. In Chapter 7, we defend the application of Evidential Pluralism to economics. We begin by scrutinising two examples: the link between the legalisation of abortion in the USA in the 1970s and the subsequent decline in the crime rates in the 1990s, and the link between unemployment and crime. These two examples highlight the roles of association studies and mechanistic studies in causal enquiry in economics. We argue that Evidential Pluralism can help us to understand the structure of causal enquiry in economics, and we discuss in more detail the role of theory in economics. Finally, we address concerns about causal pluralism (the view that there are multiple kinds of causation) in economics.

Causal enquiry is central to political science. In Chapter 8, we argue that Evidential Pluralism can account for the need for a diversity of methods in political science. We illustrate this claim by means of a case study concerning the role of resources in shaping strategies of violence during rebellions. We also argue that Evidential Pluralism captures the structure of causal enquiry in political science better than causal pluralism. Finally, we show how Evidential Pluralism coheres well with process tracing, which is a well-developed method for causal enquiry in political science, as well as multi-method large-N qualitative analysis, which is a newly emerging approach.

Causation is a key concept in the law. However, legal theorists typically presuppose that a concept of cause in the law is autonomous from that used in philosophy. This is partly because causal claims in the law are constrained by legal rules about liability, while causal claims elsewhere are not. In Chapter 9, we argue against causal autonomy in the law. We argue that a slightly modified version of Evidential Pluralism can shed light on causation in the law. This modification requires considering what we call ‘liability-tracing mechanisms’ in place of regular mechanisms. We conclude that the concept of causation in the law should not be construed as entirely autonomous from that analysed by philosophy.

Different social sciences engage in causal enquiry to a different extent. In Chapter 10, we sketch how Evidential Pluralism can be applied to a variety of other social sciences, including anthropology, psychology, demography, geography, management science and education research. We argue that Evidential Pluralism has broad scope: it applies across the social sciences, wherever there is causal enquiry.

We conclude that the main contribution of the book is a new way of thinking about causal enquiry in the social sciences. A new way of thinking can open new possibilities and provide new insights. For example, as we have argued, Evidential Pluralism can motivate new strategies for evaluating social policies.

Since the book was published, these new evaluation strategies have become the focus of a project that seeks to develop systematic review methods that accord with Evidential Pluralism (Ratnayake, 2025). In another research project, Evidential Pluralism is being applied to motivate a new approach to evidence-based law, called EBL + (Trofimov & Williamson, 2025).

## Data Availability

We do not analyse or generate any datasets, because our work proceeds within a theoretical and mathematical approach.
